# Early esophageal squamous neoplasia and the microbiome: evidence, mechanisms, and early-detection potential

**DOI:** 10.3389/fmicb.2026.1885729

**Published:** 2026-08-06

**Authors:** Qian Yang, Xiaohong Wang, Jing Cao, Chunhai Fu, Can Yang, Bojing Wang, Ping Liu, Yizhou Wang, Shu Huang, Xiaowei Tang

**Affiliations:** 1 Department of Gastroenterology, the Affiliated Hospital of Southwest Medical University, Luzhou, China; 2 Department of Gastroenterology, Xuzhou Central Hospital, Xuzhou Clinical School of Xuzhou Medical University, Xuzhou, China; 3 Department of Gastroenterology, Lianshui County People’s Hospital, Huaian, China; 4 Department of Gastroenterology, Lianshui People’s Hospital of Kangda College Affiliated to Nanjing Medical University, Huaian, China; 5 Department of Endoscopic Medicine, the Affiliated Hospital of Southwest Medical University, Luzhou, China

**Keywords:** dysbiosis, early detection, esophageal squamous cell carcinoma, HGIN, high-grade intraepithelial neoplasia, microbiome

## Abstract

Esophageal squamous cell carcinoma (ESCC) remains a major cause of cancer mortality, largely because most patients are diagnosed at an advanced stage. High-grade intraepithelial neoplasia (HGIN) represents an important and clinically actionable precancerous stage in the ESCC pathway, during which timely detection and endoscopic therapy can be curative. However, population screening with endoscopy is resource-intensive and has limited acceptability. Accumulating observational evidence suggests that ESCC and its precancerous stages are accompanied by alterations in the esophageal, oral, and tumor-associated microbiome, although the reproducibility and clinical significance of these findings vary across cohorts, sample types, and analytical methods. These microbial changes are shaped by shared risk factors, including smoking, alcohol consumption, hot beverages, poor oral hygiene, local mucosal injury and host immunity. Dysbiosis involves not only bacteria but also fungi and cross-kingdom interactions, which may be associated with barrier disruption, chronic inflammation, and malignant transformation. In this review, we summarize current evidence on the bacteriome and mycobiome across the normal squamous mucosa-HGIN-ESCC, focusing on microbial candidates, potential host–microbe mechanisms, and early-detection relevance. We also discuss saliva-based testing, non-endoscopic esophageal sampling, blood-derived microbial signals, and AI-assisted risk models. At present, microbiome-based biomarkers should be regarded as promising but not yet clinically implementation-ready tools. Further longitudinal validation, standardized methodology, and external cohort testing are required before they can be integrated into routine ESCC early-detection or risk-stratification pathways.

## 1 Introduction

Esophageal cancer (EC) remains a major global health challenge, with a particularly high incidence and mortality rate in regions such as China, where ESCC is predominant. Despite the availability of therapeutic options, the prognosis of ESCC remains poor, largely because the majority of patients are diagnosed at an advanced stage, at which point curative treatment is no longer feasible (Bray et al., 2024; Liu et al., 2024). Early detection of ESCC is critical, and HGIN, which represents a key precancerous stage, offers a potential window for intervention. HGIN is characterized by severe cytological and architectural atypia confined to the epithelium, without invasion through the basement membrane, meaning the abnormality remains confined to the epithelial layer. Terminologically, esophageal squamous intraepithelial neoplasia (ESIN) is an umbrella category encompassing low- and high-grade lesions, and HGIN denotes its high-grade category. High-grade dysplasia (HGD) is an alternative term used in some source studies for a corresponding high-grade lesion, although nomenclature and grading criteria may vary across studies. Accordingly, this review uses HGIN in the general narrative, retains HGD or ESIN when describing individual studies, and does not equate unstratified ESIN with HGIN. However, current screening methods, primarily relying on invasive endoscopy, have limitations such as cost, accessibility, and the expertise required for accurate diagnosis (Kim and Hong, 2021; Pimentel-Nunes et al., 2022). The key clinical challenge, therefore, is how to shift detection upstream—identifying patients before cancer becomes invasive, ideally before irreversible malignant progression occurs.

In high-risk regions, such as East Asia, ESCC often develops through a well-established sequence of precancerous stages, from basal cell hyperplasia and low-grade squamous intraepithelial neoplasia or dysplasia to HGIN and finally invasive carcinoma. Endoscopic screening with chromoendoscopy has proven beneficial in high-risk populations, but its widespread implementation faces practical barriers, including the invasiveness of the procedure and patient reluctance to participate. This highlights the urgent need for non-invasive, cost-effective screening alternatives that can enrich high-risk populations for endoscopy and improve the efficiency of early detection.

Recent advances in microbiome research offer exciting possibilities in this regard (Wei et al., 2022). The esophagus is home to a complex microbial community, shaped by external factors such as smoking, alcohol consumption, diet, and poor oral hygiene, all of which contribute to mucosal injury and chronic inflammation—key factors in the development of ESCC. Studies have increasingly demonstrated that microbial dysbiosis, encompassing both bacterial and fungal shifts, is associated with the transition from normal esophageal tissue to HGIN and invasive cancer (Yu et al., 2024; Gao et al., 2023). These microbial changes may contribute to ESCC pathogenesis by disrupting epithelial barrier function, sustaining inflammation, and modulating immune responses, thereby facilitating malignant transformation (Moe and Tan, 2024).

**Figure 1:**
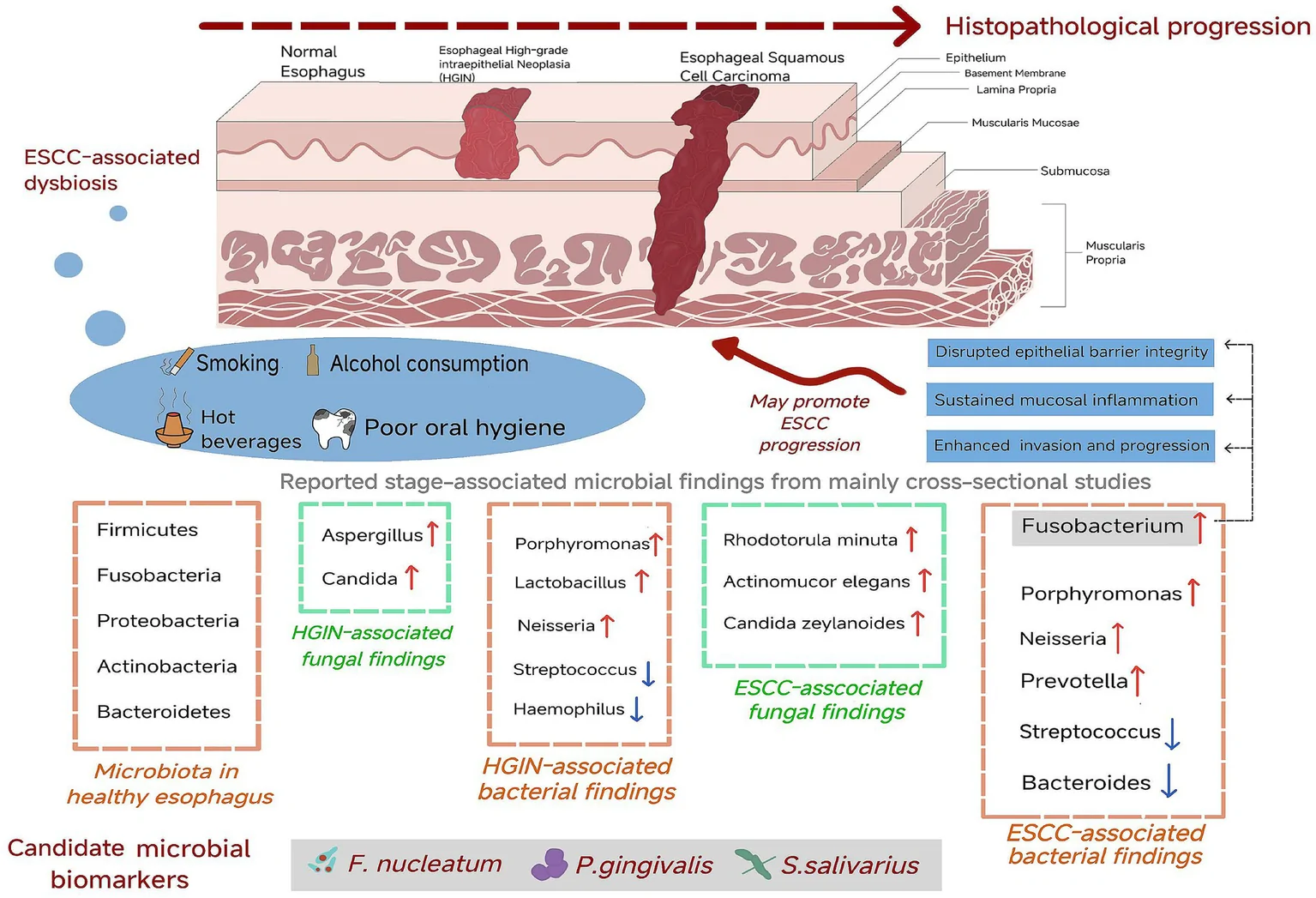
Reported microbial findings across the normal esophagus–HGIN–ESCC spectrum. The upper panel schematically illustrates the histopathological progression from normal esophageal epithelium to HGIN and ESCC. The lower panel summarizes reported stage-associated bacterial and fungal findings, major ESCC-related risk factors, potential effects on epithelial barrier integrity and inflammation, and candidate microbial biomarkers. These microbial findings are derived mainly from heterogeneous cross-sectional studies and should not be interpreted as longitudinal changes within the same individuals or as a proven microbial progression model. Upward and downward arrows indicate reported relative-abundance differences compared with the corresponding control or reference groups in individual studies. HGIN, high-grade intraepithelial neoplasia; ESCC, esophageal squamous cell carcinoma.

In this structured narrative review, we synthesize current evidence on the role of the microbiome, particularly bacterial and fungal communities, in the progression from normal esophageal mucosa to HGIN and invasive ESCC (Wei et al., 2022). We focus on the microbial shifts that occur along the continuum from normal esophageal mucosa to HGIN and invasive ESCC. These microbial changes represent potential biomarkers for early detection, offering valuable insights into disease mechanisms (Yu et al., 2024). We will explore how these microbial shifts contribute to key processes such as epithelial barrier disruption, chronic inflammation, and immune modulation, which are essential in the progression of ESCC. While we acknowledge the growing interest in non-endoscopic diagnostic methods, such as AI-assisted models and blood-based assays, the primary focus of this review remains on the microbiome as a promising tool for early detection and risk stratification. Finally, we will discuss future directions in microbiome-informed early detection strategies, emphasizing the need for rigorous validation studies to establish the clinical utility of microbiome-based biomarkers in ESCC (Figure 1).

## 2 Review methodology

### 2.1 Literature search strategy

The aim of this review was to synthesize current evidence on the microbiome in early esophageal squamous neoplasia, with emphasis on high-grade intraepithelial neoplasia (HGIN), esophageal squamous cell carcinoma (ESCC), microbial mechanisms, and early-detection potential. Relevant literature was identified through searches of PubMed/MEDLINE, Web of Science, Scopus, and Google Scholar up to 19 May 2026. The search strategy combined disease-related, microbiome-related, and clinical-translation-related terms.

The main disease-related terms included “esophageal squamous cell carcinoma,” “ESCC,” “esophageal cancer,” “esophageal squamous neoplasia,” “high-grade intraepithelial neoplasia,” “HGIN,” “dysplasia,” and “precancerous lesion.” Microbiome-related terms included “microbiome,” “microbiota,” “microflora,” “dysbiosis,” “bacteriome,” “mycobiome,” “fungal microbiome,” “oral microbiome,” “salivary microbiome,” “esophageal microbiome,” and “tumor microbiome.” Detection-related terms included “early detection,” “screening,” “biomarker,” “saliva,” “esophageal brushing,” “biopsy,” “lavage,” “wash sample,” “sponge cytology,” “Cytosponge,” “microbial cell-free DNA,” and “machine learning.” Additional targeted searches were performed for microorganisms repeatedly implicated in ESCC or esophageal precancerous lesions, including “*Fusobacterium nucleatum*,” “*Porphyromonas gingivalis*,” “*Prevotella*,” “*Streptococcus*,” “*Neisseria*,” “*Candida*,” and “*Aspergillus*.” Reference lists of relevant original articles, reviews, systematic reviews, and meta-analyses were also screened manually to identify additional studies.

### 2.2 Study selection and evidence synthesis

Studies were considered relevant if they met one or more of the following criteria: they investigated bacterial or fungal communities in ESCC, HGIN, esophageal dysplasia, or esophageal precancerous lesions; analyzed esophageal tissue, biopsy, brushing, lavage, wash samples, saliva, oral samples, stool, tumor tissue, or blood-derived microbial signals relevant to ESCC; reported microbial diversity, taxonomic shifts, interkingdom interactions, host–microbe mechanisms, or microbiome-related biomarkers; or evaluated microbiome-informed approaches for early detection, risk stratification, or screening.

Priority was given to human studies, studies involving precancerous lesions or early-stage ESCC, multicohort or prospective studies, and mechanistic studies that provided biological insight into microbial involvement in epithelial barrier disruption, inflammation, immune modulation, tumor progression, or treatment response. Studies were excluded if they focused exclusively on esophageal adenocarcinoma or Barrett’s esophagus without relevance to ESCC, did not report microbiome-related findings, were not available in English, or provided insufficient methodological or biological detail for interpretation.

Because the available literature is heterogeneous in disease stage, sample type, sequencing method, microbial kingdom analyzed, analytical pipeline, and clinical endpoint, a formal quantitative meta-analysis was not performed. Instead, the evidence was narratively synthesized and further summarized in a structured comparative table. Key studies were compared according to microbial taxa, disease stage, study design, sample type, principal findings, level of supporting evidence, and major limitations. Particular attention was paid to microbial findings that were reported across more than one study or cohort, showed consistency across biologically related sample types, were supported by mechanistic evidence, or had potential relevance to early detection and risk stratification. Evidence was interpreted according to its biological source, with oral samples, esophageal mucosal samples, tumor tissue, and blood-derived microbial signals considered separately because they represent distinct biological compartments and differ in their proximity to the esophageal lesion. ESCC-specific human and experimental findings were distinguished from mechanisms extrapolated from other cancer types, non-ESCC disease settings, or general microbial biology. Evidence status was assigned qualitatively according to study design, population and cohort coverage, disease-stage relevance, independent validation, and the availability of mechanistic support; the definitions of individual categories are provided in the footnote to Table 1.

**Table 1:** Evidence-summary table comparing representative microbial findings in ESCC and esophageal precancerous lesions.

Study/region	Study population and design	Sample size	Sample type	Main method	Alpha diversity	Key microbial findings	Evidence status	Main limitations
(Li et al., 2021); Northern China	Normal, LGD, HGD, and ESCC; cross-sectional comparison	Normal (*n* = 82) LGD (*n* = 60) HGD (*n* = 64) ESCC (*n* = 70)	Paired saliva and brushings	16S rRNA	Decreased with progression	HGD: *Lactobacillus* ↑; ESCC: *Bosea, Solobacterium, Gemella, Peptostreptococcus* ↑	Primary clinical evidence; cross-sectional	Single-region cohort; limited subgroup sample sizes
(Shijimaya et al., 2024); Japan	EC, non-EC controls; case–control study	EC (*n* = 45) non-EC (*n* = 20)	Wash samples; matched EC biopsies	16S rRNA	No significant difference	*Prevotella ↓ / Shuttleworthia ↑;* wash microbiota reflected resident flora.	Emerging clinical evidence	Small regional cohort; incomplete covariates; no follow-up;
(Chen et al., 2025); multicountry	EC vs. controls; oral microbiome meta-analysis	EC (*n* = 1,191) controls (*n* = 1,403)	Mostly saliva/oral samples	Systematic review, meta-analysis	Variable across studies	*Prevotella, Porphyromonas, and Fusobacterium ↑* in ESCC-associated oral signatures	Evidence synthesis	High interstudy heterogeneity; residual confounding; small study samples
(Yuan et al., 2025); China	HC, unstratified ESIN, and ESCC; cross-sectional tissue-based study	HC (*n* = 13) ESIN (*n* = 10) ESCC (*n* = 12)	Esophageal tissue biopsies	16S rRNA and ITS	Altered across stages	Unstratified ESIN: *Lactobacillus / Aspergillus ↑;* ESCC: *Fusobacterium, Streptococcus,* and *Alternaria ↑*	Emerging tissue-based evidence	Single-region, small cohort; no ESIN-grade stratification; lack of mechanistic validation
(Jiang et al., 2021); China	Controls, esophagitis, and ESCC; cross-sectional comparison	Controls (*n* = 21) ES (*n* = 15) ESCC (*n* = 32)	Esophageal tissue samples	16S rRNA	Different between ESCC and controls	ESCC: *Streptococcus, Actinobacillus, Peptostreptococcus, Fusobacterium, Prevotella ↑*	Primary clinical evidence; small cohort	Small group sizes; cross-sectional design
(Greathouse et al., 2024); USA/TCGA	ESCA tumor vs. non-tumor tissue; NCI-MD cohort with TCGA validation	NCI-MD (*n* = 212) TCGA RNA-seq (*n* = 172) TCGA WGS (*n* = 123)	Tumor and non-tumor esophageal tissues	16S rRNA	Variable across cohorts	*Campylobacter, Fusobacterium, Prevotella*, and *Streptococcus* ↑; *Fusobacterium–* *Prevotella* linked to immune infiltrates.	Primary multi-cohort ESCA tumor-tissue evidence; not ESCC-specific	Mixed histologies; cross-sectional design; inferred immune profiles
(Lei et al., 2023); China	Normal, EEC, and AEC; tissue validation plus mechanistic study	Normal (*n* = 12) EEC (*n* = 23) AEC (*n* = 20) ECA (*n* = 181)	Esophageal tissues; ESCC models	FISH qPCR	NA	*F. nucleatum ↑ with* ECA progression	Mechanistic and clinical tissue evidence	Small discovery cohort; single-center samples; no external or longitudinal validation

ESCC, esophageal squamous cell carcinoma; EC, esophageal cancer; ESCA, esophageal carcinoma; HC, healthy controls; LGD, low-grade dysplasia; HGD, high-grade dysplasia; HGIN, high-grade intraepithelial neoplasia; ESIN, esophageal squamous intraepithelial neoplasia; ES, esophagitis; EEC, early esophageal cancer; AEC, advanced esophageal cancer; ECA, esophageal cancer; TCGA, The Cancer Genome Atlas; WGS, whole-genome sequencing; RNA-seq, RNA sequencing; ITS, internal transcribed spacer; FISH, fluorescence in situ hybridization; qPCR, quantitative PCR; PFS, progression-free survival; NA, not applicable. ESIN is an umbrella term encompassing low- and high-grade esophageal squamous intraepithelial neoplasia, whereas HGIN denotes the high-grade category. HGD is an alternative term used in some source studies for a corresponding high-grade lesion. Original study terminology was retained, and unstratified ESIN was not treated as equivalent to HGIN. Evidence status was assigned qualitatively according to study design, cohort coverage, independent validation, and mechanistic support, and does not represent a formal GRADE-based rating. Emerging evidence refers to preliminary findings from a single, generally small or exploratory cohort; “tissue-based” specifies direct analysis of esophageal tissue. Primary clinical evidence refers to original human cohort evidence with defined disease–control or stage comparisons, whereas primary multi-cohort evidence requires replication or validation across independent cohorts. Mechanistic and clinical tissue evidence indicates concordant human tissue findings and experimental support, while evidence synthesis refers to systematic-review or meta-analytic evidence. Descriptors such as “cross-sectional,” “small cohort,” and “not ESCC-specific” indicate study limitations rather than separate evidence categories.

## 3 Microbiome landscape in HGIN and ESCC

### 3.1 Study designs and sample types

Interpreting microbiome data in the context of HGIN and ESCC begins with understanding how the data were generated, since the study design and sample type fundamentally shape what microbial signals can be observed. The vast majority of human studies to date are cross-sectional case–control designs, which are efficient at identifying microbial features that differ between disease states and controls but cannot establish temporal or causal relationships between dysbiosis and disease progression. For example, cross-sectional profiling of esophageal brushings and saliva has shown distinct bacterial signatures in ESCC patients compared with controls, including differences in *Streptococcus*, *Prevotella*, *Fusobacterium* and other taxa, yet it remains unclear whether these patterns are causative, consequential, or both (Li et al., 2021). Furthermore, the choice of control groups, such as healthy individuals versus patients with non-neoplastic esophageal conditions, can significantly influence which taxa are identified as differentially abundant, underscoring the need for careful control selection and cautious interpretation (Shijimaya et al., 2024).

In contrast, longitudinal cohort studies that track individuals over time—from health to HGIN and then to invasive ESCC—are better positioned to clarify whether microbial alterations precede, accompany, or follow neoplastic progression. Such studies are still rare in the ESCC microecology field, largely because of logistical and cost challenges associated with long-term follow-up and repeated endoscopic sampling. The scarcity of prospective data represents a major gap in the evidence needed to infer whether and how microbial shifts participate in, rather than merely reflect, carcinogenesis (Chen et al., 2025).

The type of biological sample used in microbiome studies directly defines the ecological niche represented and influences both findings and interpretation. Evidence obtained from oral samples, esophageal mucosal samples, tumor tissue, and blood-derived microbial signals should therefore be considered separately, because these sample types represent biologically distinct compartments and differ in their proximity to the esophageal lesion.

Oral samples, particularly saliva and oral swabs, primarily reflect the oral microbial ecosystem and have been explored as non-invasive proxy samples under the “oral–esophageal axis” hypothesis. Systematic reviews suggest that oral microbial shifts often involve taxa also observed in esophageal studies, indicating shared signals across niches that merit deeper investigation (Shijimaya et al., 2024). However, oral microbial associations should not be assumed to represent local esophageal dysbiosis, and their lesion specificity requires validation through systematic comparison with matched esophageal samples.

Esophageal mucosal samples include endoscopic biopsies, brushings, and lavage or wash samples, each of which captures a different component of the local esophageal environment. Endoscopic mucosal biopsies provide the most direct access to mucosa-associated microbial communities and are therefore valuable for mechanistic investigation. However, because the esophagus is a low-biomass site, biopsy samples are highly prone to contamination and require stringent quality control in DNA extraction and sequencing pipelines to avoid artefactual signals. Endoscopic brushings sample the superficial mucosal layer and generally yield more microbial DNA relative to host DNA, offering a compromise between invasiveness and data yield that may be more amenable to larger clinical studies. Esophageal lavage or wash samples reflect luminal contents and may dilute mucosa-specific signals but can still correlate with mucosal community profiles, especially when combined with matched biopsies. Accordingly, findings from biopsies, brushings, and wash samples should not be treated as directly interchangeable without considering their distinct sampling depths and ecological coverage.

Tumor-tissue studies provide lesion-proximal evidence from established ESCC and can link microbial signals to local histopathological, molecular, or immune features. However, tumor-tissue findings primarily reflect the microbial ecology of established disease and cannot by themselves demonstrate that the same microbial alterations were present during HGIN or before tumor development.

Blood-derived microbial signals, including microbial cell-free DNA, represent systemic and indirect candidate biomarkers rather than direct measurements of the oral or esophageal microbiota. Their tissue of origin, lesion specificity, and sensitivity for HGIN or early ESCC remain uncertain; therefore, these signals should be interpreted separately from findings obtained directly from esophageal mucosa or tumor tissue.

A clear understanding of these methodological considerations is essential before interpreting community shifts; with this context, we next summarize consistent microbial patterns observed along the normal–HGIN–ESCC continuum. Wherever possible, the following sections distinguish lesion-proximal findings from esophageal mucosa and tumor tissue from oral proxy signals and blood-derived candidate biomarkers. These distinctions also provide the context for interpreting the microbial patterns summarized below and in Table 1.

### 3.2 Bacterial remodeling across the normal–HGIN–ESCC continuum

The esophageal microbiome in a healthy state is characterized by low biomass and a balanced representation of bacterial phyla such as Firmicutes, Bacteroidetes, Proteobacteria, Actinobacteria, and Fusobacteria (Baba et al., 2025). Key genera, such as *Streptococcus*, *Prevotella*, and Veillonella, are commonly detected and contribute to the ecological stability of the mucosal microbiota (Zou et al., 2023). However, this equilibrium is easily disturbed by environmental factors and behaviors such as smoking, alcohol consumption, hot beverages, and poor oral hygiene; these exposures have been associated with altered esophageal microbial composition and cancer risk in human cohorts and mechanistic models, indicating that microbial dysbiosis may accompany known risk factors for ESCC development (Baba et al., 2025; Chiang et al., 2023). These factors are known to cause local tissue damage, altering the oral microbiota and indirectly affecting the esophageal microbiome by either directly translocating microorganisms or modifying the host environment. Rather than representing a single causal event, these exposures may reshape the local mucosal niche and create conditions in which dysbiosis can emerge or persist during the transition from normal mucosa to esophageal squamous intraepithelial neoplasia (ESIN), of which HGIN represents the high-grade category (Yuan et al., 2025).

Among precancerous lesions, microbial dysbiosis has been reported in studies using different histopathological terminology. Li et al. classified lesions as high-grade dysplasia (HGD), a term corresponding to a high-grade intraepithelial lesion in that study, whereas Yuan et al. analyzed an ESIN group without further grade stratification (Li et al., 2021; Yuan et al., 2025). Accordingly, findings from unstratified ESIN should be interpreted as evidence for precancerous ESIN in general rather than specifically for HGIN. Reduced *α*-diversity has been observed in some cohorts involving HGD, ESIN, and early esophageal cancer (Li et al., 2021; Yuan et al., 2025; Jiang et al., 2021). Direct esophageal studies have reported increased relative abundance of taxa such as *Prevotella*, *Lactobacillus*, *Neisseria*, and *Porphyromonas* in HGD or ESIN, whereas oral-sample studies and evidence syntheses have identified partially overlapping ESCC-associated signatures involving *Prevotella*, *Porphyromonas*, and other taxa (Li et al., 2021; Chen et al., 2025; Yuan et al., 2025; Li et al., 2023).

In oral-sample analyses, enrichment of *Prevotella*, a genus associated with inflammation and mucosal perturbation, may indicate an inflammation-associated oral signature rather than prove direct involvement in the precancerous esophageal lesion. Likewise, *Lactobacillus* spp. and *Neisseria* spp. detected in esophageal mucosal samples may be compatible with an oral–esophageal ecological connection, but these lesion-proximal findings should be distinguished from associations measured directly in saliva or oral swabs.

In direct esophageal mucosal comparisons, commensal genera such as *Streptococcus* and *Haemophilus* have been reported to be depleted in HGD, as termed in the source study, relative to healthy mucosa, consistent with a loss of commensal taxa alongside the expansion of gram-negative and inflammation-associated bacteria in dysplasia and early ESCC stages (Li et al., 2021; Zou et al., 2023). The reduction of *Streptococcus*, which has been associated with mucosal homeostasis in the upper digestive tract, may reflect weakened colonization resistance or altered mucosal ecology, rather than necessarily indicating a direct loss of protection. Together, these HGD-associated and broader ESIN-associated patterns are consistent with epithelial stress, altered barrier conditions, and low-grade mucosal inflammation, but their temporal relationship to dysplastic progression remains to be clarified.

In established ESCC, tumor-tissue studies have reported a more tumor-associated and ecologically specialized microbial profile, with taxa such as Prevotella and Neisseria enriched in some cohorts relative to adjacent non-tumor tissue (Greathouse et al., 2024). These cross-sectional tumor-tissue findings should not be interpreted as evidence that the same taxa increase longitudinally from HGD/HGIN or unstratified ESIN to ESCC. The enrichment of *Fusobacterium nucleatum* in ESCC is particularly noteworthy; it has been repeatedly identified at higher abundance in tumor tissue or other lesion-proximal samples across independent tissue-based studies, supporting its relevance as a tumor-associated microbial signal and potential biomarker rather than establishing causality by itself (Li et al., 2025; Lei et al., 2023). Separate from these human tissue associations, experimental studies have reported that *F. nucleatum* can promote proliferation- and inflammation-related cellular programs by inducing IL-32/PRTN3 expression and activating the PI3K/AKT signaling pathway in early ESCC models, linking its enrichment to biologically plausible pathways that may amplify neoplastic growth and local immune modulation (Lei et al., 2023).

In addition, the reduction of some commensal genera such as Bacteroides and other commensal taxa in ESCC relative to non-tumor controls is consistent with the loss of mucosal barrier function and the shift toward a pro-inflammatory and pro-tumorigenic environment, a pattern consistently noted in sequencing studies comparing ESCC with adjacent normal tissue (Jiang et al., 2021; Li et al., 2025).

The detection of *Porphyromonas* and *Prevotella* in HGD- or ESIN-based precancerous studies and in ESCC tumor-tissue studies indicates cross-sectional overlap across diagnostic categories, but does not demonstrate persistence or progressive enrichment within the same individuals. These bacteria, along with *Fusobacterium*, may form part of a tumor-associated ecological network linked to immune modulation, tissue remodeling, and invasion-related pathways. However, whether these taxa actively drive the transition to invasive ESCC, expand secondarily within an altered tumor microenvironment, or participate in both processes remains unresolved.

Overall, heterogeneous studies using oral samples, esophageal mucosal samples, and tumor tissue have reported partially overlapping but non-interchangeable bacterial signals in HGD/HGIN, broader ESIN categories, and ESCC. Direct esophageal and tumor-tissue studies suggest reduced diversity, enrichment of oral- and inflammation-associated taxa such as *Prevotella*, *Porphyromonas*, and *Fusobacterium*, and depletion of commensal taxa such as Streptococcus and *Haemophilus*, whereas oral-sample findings should be interpreted as proxy signatures requiring validation against matched esophageal samples. These changes are consistent with a mucosal environment characterized by barrier perturbation, chronic inflammation, and immune modulation, but they should not be interpreted as definitive evidence of causality.

### 3.3 Fungal alterations and cross-kingdom interactions in HGIN and ESCC

Compared with the bacterial microbiota, the esophageal fungal community (mycobiome) remains far less explored, largely due to its lower biomass, higher susceptibility to contamination, and limited taxonomic resolution of current reference databases. Pan-cancer analyses have shown that fungi can display cancer-type-specific community structures and organized interactions with bacteria and host immunity across multiple tumor types (Ding et al., 2025; Zong et al., 2023; Narunsky-Haziza et al., 2022; Dohlman et al., 2022). However, these studies provide a broader conceptual framework rather than direct ESCC-specific mechanistic evidence, and their findings should not be assumed to operate identically in esophageal squamous neoplasia. In the healthy esophagus, fungal taxa such as Candida and Aspergillus are consistently detected at low abundance and are generally considered commensal constituents of the upper gastrointestinal ecosystem (Zong et al., 2023). Under physiological conditions, fungal growth is constrained by intact epithelial barriers, mucosal immunity, and competitive or antagonistic interactions with dominant bacterial genera, including *Streptococcus*. These ecological concepts are derived mainly from general upper gastrointestinal microbial biology rather than direct experimental studies in ESCC. Importantly, many established ESCC risk factors—such as tobacco smoking, alcohol consumption, thermal injury from hot beverages, and poor oral hygiene—are capable of perturbing this equilibrium by reshaping the local physicochemical and immunological niche, thereby predisposing the fungal community to ecological imbalance rather than necessarily indicating a direct carcinogenic role.

In esophageal precancerous lesions, emerging data suggest that fungal alterations may already be detectable, although the available evidence is derived mainly from broadly defined or unstratified ESIN rather than specifically confirmed HGIN. Their patterns appear more subtle and heterogeneous than those observed for bacteria. Studies specifically examining HGIN-associated mycobiome changes remain scarce, and cross-cohort consistency is modest, reflecting both technical constraints and biological variability (Ding et al., 2025; Narunsky-Haziza et al., 2022). Nonetheless, signals involving Candida and Aspergillus have been reported in subsets of premalignant esophageal lesions, including ESIN cohorts without further histological grade stratification, suggesting that fungal changes may accompany early mucosal stress or inflammation in selected contexts. From a functional perspective, this observation is biologically plausible. Certain Candida species possess the ability to undergo yeast-to-hyphae transition, adhere to damaged epithelium, and compromise barrier integrity, while also shaping local immune responses, including Th17-skewed inflammation. However, these biological properties are established mainly from general Candida biology and non-ESCC experimental settings and have not been directly demonstrated as mechanisms driving ESIN or HGIN progression. Such effects could interact with an inflamed or injured mucosal microenvironment, but direct evidence that fungal alterations drive the development or progression of ESIN, including its high-grade HGIN category, remains limited. Indirect epidemiological support comes from patients with achalasia, in whom esophageal *Candida* infection has been associated with an increased risk of esophageal cancer (Guo et al., 2025; Wang et al., 2023). Nevertheless, this association arises from a distinct high-risk disease setting and should not be regarded as direct evidence of a causal role for Candida in HGIN or ESCC.

In advanced ESCC, fungal dysbiosis has been reported to become more apparent in some tissue-based studies, coinciding with the establishment of a tumor-associated microbial niche. ESCC-specific tissue profiling has demonstrated distinct fungal community structures and reorganization of bacteria–fungi ecological networks in established tumors (Rao et al., 2023). This represents direct disease-specific observational evidence, although it does not establish causal interactions. Notably, several fungal species—including *Rhodotorula minuta*, *Actinomucor elegans*, and *Candida zeylanoides*—have been reported to be selectively enriched in ESCC tissues and to carry potential discriminatory value in specific study settings (Liang et al., 2025). By contrast, proposed mechanisms involving fungal metabolite-mediated genotoxic or epigenetic effects, epithelial disruption by invasive fungal morphologies, and persistent tumor-promoting inflammation are derived largely from pan-cancer research or general fungal biology. These mechanisms have not yet been directly validated in ESCC-specific experimental systems. Therefore, although the observed fungal signatures provide disease-specific associations, their functional contributions remain largely hypothesis-generating and require experimental and longitudinal validation. Equally important is the potential for cross-kingdom cooperation. In ESCC, current direct evidence is based mainly on tissue-level co-abundance patterns and inferred ecological networks rather than experimentally demonstrated bacteria–fungi interactions (Rao et al., 2023). Fungi may provide structural or metabolic support for anaerobic bacteria enriched in tumors, while bacteria may in turn modulate oxygen availability or nutrient landscapes favorable to fungal persistence. However, these proposed forms of metabolic cooperation, biofilm organization, and niche modification are extrapolated primarily from broader microbial ecology and other disease contexts. Their occurrence and functional importance in ESCC remain to be established. Such interactions may be relevant because fungi can influence biofilm architecture, local oxygen gradients, metabolite availability, and epithelial immune responses, whereas bacteria can shape the inflammatory and nutritional context that permits fungal persistence. Such interkingdom synergy offers a coherent ecological explanation for the convergence of microbial dysbiosis, chronic inflammation, and enhanced invasion observed in advanced disease. However, most bacteria–fungi interaction networks in ESCC are currently inferred from co-abundance or tissue-profiling data rather than experimentally validated interaction models. Therefore, cross-kingdom findings should be viewed as an emerging framework for understanding tumor-associated mucosal ecology, not as established diagnostic or causal evidence.

Overall, available studies suggest that fungal profiles differ across non-malignant mucosa, precancerous ESIN, and established ESCC, but they do not establish a longitudinal mycobiome trajectory. Fungal signals are typically low and constrained in non-malignant mucosa, appear more variable in unstratified ESIN, and become more disease-associated in established ESCC, whereas evidence specific to HGIN remains insufficient. In established ESCC, where certain taxa show preliminary discriminatory potential in limited cohorts (Ding et al., 2025; Zong et al., 2023). Importantly, ESCC-specific mycobiome evidence remains predominantly descriptive and tissue-based, whereas many proposed biological mechanisms are extrapolated from pan-cancer studies, non-ESCC disease settings, or general fungal biology. At the same time, the current body of evidence remains less mature than that for bacteria. Low fungal biomass, incomplete reference databases, and substantial methodological heterogeneity (sampling sites, ITS regions, sequencing depth, and bioinformatic pipelines) all limit cross-study comparability and make it difficult to distinguish true biological shifts from technical effects (Ding et al., 2025; Zong et al., 2023). As a result, most mycobiome findings in ESCC should still be interpreted as association-level signals rather than definitive causal contributors to carcinogenesis. Thus, the value of mycobiome research at present may lie less in immediate diagnostic application and more in clarifying how fungi, bacteria, and the injured esophageal mucosa interact within a broader tumor-associated ecological network.

To facilitate comparison across heterogeneous studies and to distinguish relatively well-supported findings from emerging or preliminary observations, representative microbial findings in ESCC and esophageal precancerous lesions are summarized in Table 1. The level of support was assigned qualitatively for narrative comparison rather than formal evidence grading (Table 1).

## 4 Potential molecular mechanisms of key microbial candidates in ESCC

This section focuses primarily on bacterial candidates for which ESCC-specific clinical or experimental evidence is available; fungal mechanisms are discussed separately in Section 3.3 and remain more dependent on extrapolation from pan-cancer studies and general fungal biology.

**Figure 2:**
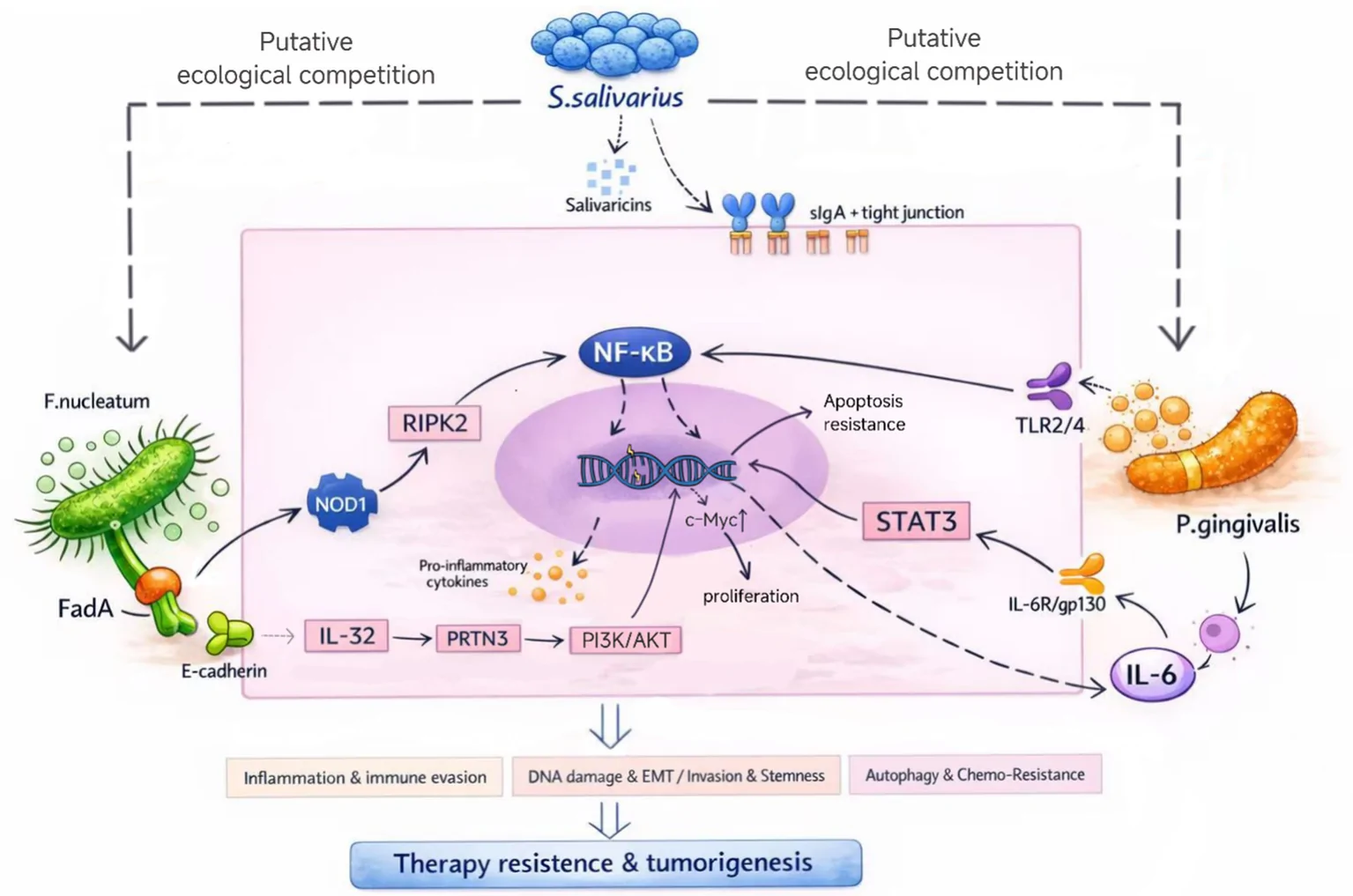
Potential microbial mechanisms involved in ESCC progression. The schematic summarizes the reported and proposed interactions of *Fusobacterium nucleatum*, *Porphyromonas gingivalis*, and *Streptococcus salivarius* with inflammatory, proliferative, immune-related, and treatment-resistance pathways in ESCC. The pathways involving *F. nucleatum* and *P. gingivalis* are supported mainly by tumor-tissue observations and experimental models, whereas the proposed role of *S. salivarius* is based largely on oral or salivary associations. Gray elements and dashed lines indicate hypothetical pathways or interactions that have not been fully validated.

Several key microorganisms implicated in ESCC, including *Fusobacterium nucleatum*, *Porphyromonas gingivalis*, and *Streptococcus salivarius*, have been linked to epithelial barrier disruption, chronic inflammation, immune modulation, and activation of tumor-promoting signaling pathways (Figure 2). However, the sources and maturity of evidence differ substantially among these microorganisms. Mechanistic evidence for *F. nucleatum* and *P. gingivalis* is derived mainly from ESCC tumor tissues and experimental models, whereas evidence for *S. salivarius* is primarily based on salivary or oral microbial associations. Blood-derived microbial signals are not considered direct mechanistic evidence in this section and are discussed separately in Section 5.

### 4.1 Fusobacterium nucleatum

Human evidence for *Fusobacterium nucleatum* (Fn) is derived mainly from analyses of ESCC tumor tissues, in which Fn abundance has been associated with adverse tumor-related features. Experimental cell and animal studies have subsequently identified biologically plausible pathways through which this organism may influence ESCC cell behavior and the tumor microenvironment. Fn can invade ESCC cells and activate innate immune sensors such as NOD1/RIPK2, leading to nuclear translocation of NF-κB and upregulation of inflammation-related genes; in experimental models, this pathway was associated with enhanced ESCC cell growth and tumor progression (Nomoto et al., 2022). This finding provides mechanistic support for a link between Fn and inflammatory signaling in ESCC, but it does not by itself establish that Fn initiates human ESCC.

In addition to canonical NF-κB signaling, Fn increases expression of IL-32 and PRTN3, triggering the PI3K/AKT pathway, a central axis associated with ESCC cell proliferation and tumor growth *in vitro* and *in vivo* (Lei et al., 2023). Lei et al. further reported enrichment of Fn in early esophageal cancer tissues and proposed that the IL-32/PRTN3–PI3K/AKT axis may help explain how Fn is linked to early tumor-promoting cellular programs. Thus, this evidence combines lesion-proximal tissue detection with experimental mechanistic validation, rather than relying on oral microbial profiling alone.

Beyond proliferation-related signaling, Fn engagement with host cells has been shown to influence the secretory phenotype of senescent ESCC cells following chemotherapy. Specifically, Fn was reported to enhance chemotherapy-induced senescence-associated secretory phenotype (SASP) secretion through activation of the DNA damage response pathway, thereby contributing to ESCC progression and chemoresistance in experimental settings (Zhang et al., 2023). This work links Fn to therapy-associated tumor microenvironmental remodeling, although its relevance to untreated HGIN-to-ESCC progression remains less directly established.

Fn has also been linked more directly to immune evasion in ESCC experimental models. Intracellular *F. nucleatum* may attenuate antitumor immunity by promoting Fn-Dps–ATF3-dependent PD-L1 transcription and by suppressing T-cell proliferation and cytokine secretion, thereby reducing the efficacy of PD-L1 blockade in experimental ESCC models (Li et al., 2023). However, whether this mechanism operates during HGIN or the earliest stages of ESCC development remains unknown.

Recent multi-omics analyses of ESCC tissues have also associated Fn-related microbial signals with metabolic, transcriptomic, and immune-related features of the tumor microenvironment (Li et al., 2025; Zhang et al., 2025). For example, available multi-omics evidence supports the possibility that Fn-related microbial signals may interact with immune-cell programs, including macrophage-associated phenotypes, but these findings remain largely association-based and require additional functional validation (Zhang et al., 2025). Therefore, although selected Fn-mediated immune-evasion mechanisms have been experimentally demonstrated in ESCC models, the broader relationships between Fn, immune-cell composition, and the human ESCC microenvironment remain incompletely validated.

Collectively, tumor-tissue observations and experimental studies support several plausible Fn-associated mechanisms in ESCC, including NOD1/RIPK2/NF-κB-mediated inflammatory signaling, IL-32/PRTN3–PI3K/AKT-associated proliferative signaling, chemotherapy-induced SASP enhancement, and possible immune-microenvironment modulation. However, whether Fn acts as an initiating factor in early esophageal neoplasia, an amplifier of inflammation and tumor-associated signaling, or a microorganism that preferentially expands within an already altered mucosal or tumor niche remains unresolved. Thus, Fn should currently be regarded as a well-supported microbial candidate and potential biomarker in ESCC, rather than a definitively proven causal driver of malignant transformation.

### 4.2 Porphyromonas gingivalis

*Porphyromonas gingivalis* (Pg), a major periodontal pathogen, has been detected in ESCC tissues and has been associated with tumor progression and clinical outcome in several studies. These clinical observations are derived mainly from tumor-tissue analyses in patients with established ESCC and should be distinguished from Pg signals detected in oral or salivary samples. However, its role in ESCC should be interpreted as a mechanistically plausible microbial candidate rather than a proven causal driver of esophageal carcinogenesis.

Mechanistically, Pg infection has been shown to regulate ESCC-related signaling pathways in experimental models. Pg can upregulate miR-194, leading to downregulation of GRHL3 and PTEN and subsequent activation of the Akt pathway. In this setting, Pg infection was associated with increased ESCC cell proliferation, migration, and invasion, suggesting that Pg may modulate tumor-cell behavior under defined experimental conditions (Liang et al., 2020). More recent evidence also suggests that Pg-induced autophagy may aggravate ESCC progression through a YAP/TAZ–miR-21-5p axis, further linking Pg to epithelial stress responses and tumor-associated signaling (Qi et al., 2025). In addition, *P. gingivalis* infection increased IL-6 production and promoted epithelial–mesenchymal transition and the recruitment of myeloid-derived suppressor cells in experimental ESCC models, providing a potential connection between microbial infection and tumor-promoting immune remodeling (Chen et al., 2021).

In clinical tumor-tissue studies, Pg positivity has been associated with more aggressive ESCC phenotypes, poorer prognosis, and reduced response to neoadjuvant chemotherapy (Yoneya et al., 2025; Gao et al., 2021). These findings support the potential clinical relevance of Pg, but they remain largely association-based and mainly involve established disease rather than the earliest stages of malignant transformation. Moreover, Pg-related signals may be influenced by oral hygiene, periodontal disease, smoking, alcohol exposure, and other ESCC risk factors. Accordingly, the presence of Pg in tumor tissue does not establish whether oral colonization preceded esophageal neoplasia or whether Pg accumulated secondarily within the tumor-associated environment.

Overall, Pg provides an important example of a bacterium with both tumor-tissue clinical associations and experimental mechanistic support in ESCC. At present, Pg is best regarded as a candidate microbial contributor or modifier of the esophageal tumor-associated mucosal environment, rather than a definitively proven initiator of ESCC.

### 4.3 Streptococcus salivarius

Unlike Fn and Pg, *Streptococcus salivarius* (Ss) is generally considered a commensal of the oral and upper digestive tract, and direct mechanistic evidence for its role in ESCC remains scarce. Available human evidence for Ss is derived primarily from oral and salivary samples rather than direct esophageal mucosal or tumor-tissue analyses. Ss has been reported at higher levels in saliva from ESCC patients and has been evaluated as part of a salivary microbial marker panel, suggesting that it may serve as an association-level marker of upper aerodigestive dysbiosis rather than a driver of oncogenic signaling per se (Wei et al., 2022). Streptococci, including Ss, can produce bacteriocin-like inhibitory substances that influence competitive interactions within the microbial community and can modulate local immune responses, but these functions have not been clearly linked to ESCC-specific tumor-promoting pathways. Consistent with this interpretation, concrete evidence linking *Ss* to specific pro-tumor signaling pathways in ESCC is lacking; most studies to date have focused on descriptive microbiome profiling rather than molecular mechanisms (Su et al., 2024).

Therefore, Ss is best viewed as an indicator taxon of microbial imbalance or altered oral–esophageal ecology in patients with ESCC, rather than as a tumor-tissue microbial signal and its mechanistic contributions—if any—remain to be established (Park and Lee, 2020). Although some community-level effects, such as biofilm organization or niche competition, may indirectly influence mucosal inflammation, current evidence does not support classifying Ss as a proven microbial driver of ESCC (Zhou et al., 2021).

Overall, Ss should currently be interpreted primarily as a salivary dysbiosis-associated marker or possible ecological modifier, rather than a causal contributor to malignant transformation. Its potential diagnostic or risk-stratification value requires validation in independent cohorts and against matched esophageal mucosal and tumor-tissue samples, while its biological role in bacteria–mucosa interactions remains largely hypothetical.

## 5 Complementary early-screening approaches beyond conventional endoscopy

While understanding microbial mechanisms is crucial, the clinical translation of these insights relies on incorporating microbial signals into practical, scalable screening workflows. Endoscopy remains the gold standard for diagnosing esophageal squamous cell carcinoma (ESCC), but its invasive nature and resource demands make it unsuitable as a primary screening tool for the general population. Therefore, there is a critical need for complementary, non-invasive tools that can enrich high-risk populations and triage them for confirmatory endoscopy. At the current stage, microbiome-based biomarkers should be viewed as adjunctive risk-enrichment tools rather than replacements for diagnostic endoscopy or histopathological confirmation. Their most plausible near-term role is to help identify individuals who may benefit from endoscopic assessment, closer surveillance, or combined risk-stratification strategies. In this context, microbiome-based approaches may contribute to three clinically relevant steps: prioritizing individuals for endoscopy, improving the recognition of early or precancerous disease when combined with other diagnostic information, and informing future surveillance or intervention strategies after risk classification.

Among the most promising complementary approaches are non-endoscopic esophageal sampling, saliva-based microbial testing, blood-derived microbial cell-free DNA (mcfDNA), and AI-based risk prediction. These approaches interrogate biologically distinct compartments: non-endoscopic esophageal sampling provides lesion-proximal signals, saliva provides an oral proxy signal, and blood-derived microbial cell-free DNA represents an indirect systemic signal.

Non-endoscopic esophageal sampling, exemplified by sponge-based cytology devices, provides a practical compromise between accessibility and data yield. These tools can collect esophageal cells and potentially microbial DNA without conventional endoscopy, offering a scalable approach for population-level triage. Studies have shown that sponge-based sampling devices can identify clinically significant esophageal pathology with high sensitivity, and such approaches have been evaluated as non-invasive triage tools for esophageal cancer screening (Gao et al., 2023). If validated for microbial readouts, such devices could help bridge the gap between community-level screening and endoscopy by identifying individuals with abnormal cytological, molecular, or microbial signals who should be prioritized for confirmatory examination. However, the evidence supporting microbiome readouts from non-endoscopic esophageal sampling remains less mature than that for cytological or imaging-based endpoints. Their ability to reproducibly detect HGIN or early ESCC requires further validation. Microbial signals obtained using these devices should therefore be interpreted as esophagus-derived local signals rather than as oral or blood-based biomarkers.

Saliva-based microbial testing offers another non-invasive and repeatable screening strategy. By analyzing the oral microbiome, specifically through a targeted panel of bacteria such as *Fusobacterium nucleatum*, *Porphyromonas gingivalis*, and *Streptococcus sali*var*ius*, saliva may provide a convenient medium for large-scale initial risk assessment. These microbial markers have demonstrated the ability to differentiate ESCC cases from controls, with studies reporting good discriminatory performance when combined with clinical risk factors (Yu et al., 2024; Li et al., 2024). The ease of sampling and the potential for longitudinal monitoring make this approach particularly attractive for population-wide screening programs. In future screening workflows, saliva-based microbial panels may be most useful as a low-burden first-line risk-enrichment tool, particularly when integrated with age, family history, smoking, alcohol consumption, dietary exposures, oral health status, and regional ESCC risk. Individuals with persistently high-risk microbial profiles could then be referred for endoscopic assessment or closer follow-up, whereas low-risk individuals might avoid unnecessary invasive procedures. Nevertheless, saliva primarily reflects the oral microbial ecosystem and may be influenced by oral hygiene, periodontal status, diet, smoking, alcohol consumption, antibiotic exposure, and other host or environmental factors. Accordingly, salivary microbial signatures should not be considered equivalent to microbiota detected directly in esophageal mucosa or tumor tissue. Therefore, saliva-based microbial panels should currently be interpreted as promising tools for risk stratification or pre-endoscopic triage, rather than stand-alone diagnostic tests.

Blood-based liquid biopsy approaches, particularly circulating mcfDNA, are emerging as a novel tool for cancer detection. The detection of microbial DNA in blood samples may provide systemic information related to tumor-associated microbial niches or host–microbe interactions. Although this field is still in its infancy for ESCC, recent studies have shown promise in using mcfDNA for early cancer detection across various tumor types, suggesting it could be expanded to esophageal cancer as well (Zong et al., 2023). Unlike microbial profiles obtained directly from esophageal mucosa or tumor tissue, blood-derived mcfDNA does not currently provide definitive information about the anatomical origin of the detected microbial signals. For ESCC, microbial mcfDNA remains exploratory, with unresolved issues related to low microbial abundance, contamination, tissue-of-origin inference, and sensitivity for HGIN or early ESCC. Thus, mcfDNA-based microbial biomarkers may be more appropriately considered a future complementary layer within multi-analyte detection platforms rather than a currently established screening modality for ESCC. If combined with host-derived methylation, mutation, inflammatory, or protein markers, microbial mcfDNA may eventually help refine early diagnosis by adding a microbial dimension to multi-analyte cancer detection models.

AI and machine learning (ML) may help optimize risk prediction and triage workflows. By integrating microbiome data with clinical variables, oral health status, lifestyle exposures, imaging findings, and other omics data, AI/ML models may refine the identification of individuals at increased risk for ESCC. This can help prioritize individuals for endoscopic examination, reducing the number of unnecessary procedures while improving the efficiency of screening workflows (Gao et al., 2023). Additionally, AI-assisted image analysis in endoscopy has shown promise in reducing missed diagnoses of early ESCC and precancerous lesions (Gehrung et al., 2021). However, microbiome-based ML models remain vulnerable to overfitting, batch effects, and population-specific signals, and therefore require external validation and calibration against clinically meaningful endpoints before clinical use.

Together, these approaches support a potential “screen–enrich–confirm” strategy for ESCC detection. In this framework, microbiome-based assays would function as first-line enrichment or risk-stratification tools, whereas endoscopy and histopathology would remain the confirmatory standards. Saliva, non-endoscopic esophageal sampling, blood-based microbial signals, and AI-assisted models may each contribute different layers of information. These signals are complementary but should not be interpreted as interchangeable because they differ in biological source, proximity to the esophageal lesion, and current level of validation. By integrating microbial signatures with clinical risk factors, cytology, imaging, and histopathology, this framework could help optimize endoscopic triage, improve confidence in detecting HGIN or early ESCC, and guide tailored surveillance or intervention strategies for high-risk individuals. At present, microbiome-based biomarkers are promising but not yet implementation-ready for routine ESCC screening. Their future clinical value will depend on reproducible performance across populations, standardized protocols, confounder control, external validation, health-economic evaluation, and integration into feasible screening pathways in high-risk regions.

## 6 Major limitations of current evidence

Despite increasing evidence linking microbial dysbiosis to ESCC and its precancerous stages, several important limitations should be considered when interpreting current findings.

First, most available human studies are cross-sectional or case–control in design. Such studies are useful for identifying microbial differences between ESCC, precancerous lesions, and control samples, but they cannot determine whether dysbiosis precedes malignant transformation, results from tumor-associated ecological changes, or reflects both processes. This limitation is particularly important in the context of HGIN and early ESCC, where distinguishing causal microbial drivers from secondary microbial alterations is essential for biomarker development and preventive intervention.

Second, the esophagus is a low-biomass microbial environment, making contamination a major methodological concern. Reagent-derived contaminants, environmental DNA, oral carryover, and contamination introduced during endoscopic sampling may disproportionately influence microbial profiles, particularly in biopsy-based studies. This issue is especially relevant for fungal microbiome analysis, where fungal biomass is often lower than bacterial biomass and current reference databases remain relatively incomplete. These methodological vulnerabilities make it difficult to distinguish true mucosa-associated microbial signals from technical artifacts in some studies. Future investigations may therefore benefit from more explicit reporting of contamination-control procedures, the use of appropriate negative controls and mock communities where feasible, and contamination-aware analytical approaches to improve the interpretability and reproducibility of low-biomass esophageal microbiome data.

Third, substantial heterogeneity across sample types, sequencing methods, and analytical pipelines limits the comparability of current studies. Different specimens, including esophageal biopsies, brushings, lavage or wash samples, saliva, oral swabs, stool, tumor tissue, and blood-derived microbial DNA, capture distinct microbial niches and may therefore yield different disease-associated signals. In parallel, variation in sequencing strategies, such as 16S rRNA sequencing, ITS sequencing, shotgun metagenomics, and targeted microbial assays, can influence taxonomic resolution and the detection of bacterial or fungal taxa. Differences in DNA extraction protocols, primer selection, sequencing depth, taxonomic classifiers, reference databases, contamination filtering, normalization methods, and statistical models further complicate cross-study comparisons. These methodological differences may partly explain why some microbial signatures are not consistently reproduced across cohorts.

Fourth, confounding factors are not uniformly controlled across studies. Smoking, alcohol consumption, hot beverage intake, diet, oral hygiene, periodontal disease, antibiotic exposure, proton pump inhibitor use, comorbidities, geographic background, socioeconomic status, and tumor stage can all shape microbial communities and are also related to ESCC risk. Without careful adjustment for these factors, it is difficult to determine whether specific microbial taxa are independently associated with esophageal carcinogenesis or simply reflect shared risk exposures.

Fifth, longitudinal validation remains limited. Few studies have followed high-risk individuals over time from normal mucosa to dysplasia, HGIN, and invasive ESCC. As a result, the temporal stability, predictive value, and causal relevance of candidate microbial biomarkers remain uncertain. In particular, it remains difficult to determine whether the reported microbial alterations precede neoplastic progression, accompany early mucosal transformation, or arise as secondary changes after tumor development. This limitation reduces the current ability to distinguish potential microbial drivers from disease-associated microbial consequences.

Overall, current studies provide important evidence that microbial dysbiosis is associated with ESCC and its precancerous stages. However, the strength of this evidence is limited by cross-sectional study designs, low-biomass contamination, methodological heterogeneity, residual confounding, and insufficient longitudinal validation. These limitations should be considered when interpreting reported microbial signatures, especially in relation to causality, biomarker robustness, and clinical applicability.

## 7 Summary and outlook

Microbial alterations have been increasingly reported across non-malignant esophageal mucosa, precancerous lesions, and established ESCC. These stage-associated patterns are derived predominantly from heterogeneous cross-sectional studies and should not be interpreted as demonstrating a longitudinal microbial trajectory or causal disease progression. Throughout this review, we have highlighted reported bacterial and fungal associations across these disease categories and their potential biological relevance to host programs governing barrier function, inflammation, and immune tone (Annavajhala et al., 2020).

Mechanistically, the field is moving from “who is there” to “what they do.” For example, intratumoral *Fusobacterium nucleatum* can behave as an intracellular pathogen and dampen antitumor immunity in ESCC, offering a plausible mechanistic link between a taxon-level signal and immune escape biology (Li et al., 2023). Likewise, periodontal pathogens such as *Porphyromonas gingivalis* are being tied to actionable cancer-relevant pathways; recent work suggests that *P. gingivalis*–induced autophagy may aggravate ESCC progression through a YAP/TAZ–miR-21-5p axis, providing a possible route from colonization pressure to epithelial reprogramming (Qi et al., 2025). Beyond single taxa, host–microbe inflammatory circuits are also coming into sharper focus: gut-derived Bacteroides/LPS–TLR4–MyD88–NF-κB signaling has been implicated in invasion/metastasis phenotypes in ESCC models, underscoring how microbial products may amplify pro-tumor inflammation and EMT-like programs (Wu et al., 2024). However, most mechanistic evidence remains derived from experimental models or association-based human studies, and direct causal roles in human ESCC initiation or progression remain to be established.

Clinically, microbiome signals remain attractive because they are measurable, dynamic, and potentially modifiable. Nevertheless, their clinical use in ESCC early detection and risk stratification remains exploratory. Translation will depend on prospective validation, careful control of confounders (diet, smoking/alcohol, oral health, antibiotics/PPIs), and harmonized protocols for sampling, sequencing, and bioinformatics so that “signatures” are reproducible across cohorts and geographies (Annavajhala et al., 2020). Another practical frontier is whether microbiome states track clinically meaningful endpoints—progression risk, recurrence, and treatment response. In this context, *P. gingivalis* infection has been associated with more aggressive behavior and reduced chemotherapy efficacy in ESCC, suggesting that microbial readouts may eventually complement, rather than replace, established clinical and pathological risk factors (Gao et al., 2021).

Looking ahead, the most useful next step is integration: pairing mechanistic microbial biology with host immune/metabolic readouts and robust longitudinal sampling across the normal–HGIN–ESCC continuum. Rather than implying clinical readiness, current evidence supports a more cautious view: the microbiome is a promising source of biological insight and candidate biomarkers, but its role in ESCC prevention, early detection, and precision management will require longitudinal validation, external cohort testing, standardized methodology, and demonstration of added clinical value. If stable and generalizable microbial patterns can be linked to host pathways and clinically meaningful endpoints, microbiome-informed approaches may eventually become a useful adjunct to existing ESCC screening and management strategies (Li et al., 2023; Qi et al., 2025; Gao et al., 2021; Wu et al., 2024).
